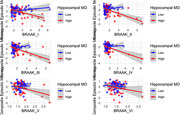# Hippocampal Microstructure as a Measure of Cognitive Resilience to Early Braak Staged Tau PET burden in Dementia‐free older adults

**DOI:** 10.1002/alz70862_110054

**Published:** 2025-12-23

**Authors:** Daniel D. Callow, Nisha Rani, Kylie H. Alm, Corinne Pettigrew, Anja Soldan, Michael I Miller, Marilyn S. S. Albert, Arnold Bakker

**Affiliations:** ^1^ Johns Hopkins University School of Medicine, Baltimore, MD USA; ^2^ Department of Neurology, Johns Hopkins University School of Medicine, Baltimore, MD USA; ^3^ Johns Hopkins University Whiting School of Engineering, Baltimore, MD USA; ^4^ Department of Psychiatry and Behavioral Science, Johns Hopkins University School of Medicine, Baltimore, MD USA

## Abstract

**Background:**

Cognitive resilience, the ability to maintain cognitive function despite neuropathological burden, is increasingly recognized as a key determinant of clinical outcomes in Alzheimer’s disease (AD). While hippocampal volume is a well‐established marker of neurodegeneration, hippocampal microstructure, assessed via diffusion‐weighted imaging (DWI), may provide a more sensitive measure of tissue integrity and cognitive function in the context of AD‐related pathology.

**Method:**

This study investigated hippocampal microstructure as a potential measure of cognitive resilience, by examining its role in moderating the relationship between in vivo tau burden (measured by positron emission tomography [PET]) and cognitive performance across multiple domains. The study included 192 participants without dementia (14 with mild cognitive impairment [MCI]) from the BIOCARD Study (mean age = 68), of whom 52 (27%) were Aβ positive (measured via PET). Multiple linear regression analyses tested whether hippocampal mean diffusivity (MD), a DWI‐derived metric, moderated the associations between tau accumulation on PET (within subregions defined as Braak stage I‐V) and global and domain‐specific cognitive composite scores (episodic memory, executive function, visuospatial abilities, and language), while controlling for age, sex, education, APOE genetic risk, amyloid positivity status, and hippocampal volume.

**Result:**

Lower hippocampal MD (indicating better microstructural integrity) significantly attenuated the negative association between greater tau burden in Braak stage I‐V and poorer global cognition (interaction *p*‐values < 0.006). Similar moderating effects were observed for episodic memory and language, but not for executive function or visuospatial abilities. In contrast, hippocampal volume did not moderate the relationship between tau burden and global or subdomain specific cognition (all *p*‐values > 0.150).

**Conclusion:**

These findings suggest that better hippocampal microstructure may be a sensitive biomarker of cognitive resilience, by partially mitigating the adverse relationship between greater tau burden and poorer cognition. Unlike volumetric measures, diffusion‐weighted imaging metrics may offer valuable insights into brain mechanisms and hold promise as outcome measures for early interventions to preserve cognitive function in AD.